# Perceptions, barriers and enablers on salt reduction in the out-of-home sectors in Malaysia (MySaltOH) from the perspective of street food vendors, caterers and consumers

**DOI:** 10.1017/S136898002300277X

**Published:** 2023-12-15

**Authors:** Zainorain Natasha Zainal Arifen, Hasnah Haron, Suzana Shahar, Zaliha Harun, Viola Michael, Yee Xing You, Zahara Abdul Manaf, Hazreen Abdul Majid, Yook Chin Chia, Feng J He, Mhairi Karen Brown, Graham A MacGregor

**Affiliations:** 1 Nutritional Sciences Programme, Centre for Healthy Ageing and Wellness, Faculty of Health Sciences, Universiti Kebangsaan Malaysia, Jalan Raja Muda Abdul Aziz, Kuala Lumpur 50300, Malaysia; 2 Dietetic Programme, Centre for Healthy Ageing and Wellness, Faculty of Health Sciences, Universiti Kebangsaan Malaysia, Jalan Raja Muda Abdul Aziz, Kuala Lumpur 50300, Malaysia; 3 Centre for Population Health, Department of Social and Preventive Medicine, Faculty of Medicine, University of Malaya, Kuala Lumpur 50603, Malaysia; 4 Department of Medical Sciences, School of Medical and Life Sciences, Sunway University, Petaling Jaya, Selangor Darul Ehsan 47500, Malaysia; 5 Wolfson Institute of Preventive Medicine, Barts and The London School of Medicine and Dentistry, Queen Mary University of London, London EC1M 6BQ, UK

**Keywords:** Perceptions, Barriers, Enablers, Salt reduction, Out-of-home sectors, Food vendors, Caterers, Consumers

## Abstract

**Objective:**

To explore the perspectives, barriers and enablers on salt reduction in out-of-home sectors in Malaysia among street food vendors, caterers and consumers.

**Design:**

A qualitative study involving twenty-two focus group discussions and six in-depth interviews was conducted, recorded and transcribed verbatim. An inductive thematic analysis approach was employed to analyse the data.

**Setting:**

Two in-depth interviews and twenty-two focus group discussions were conducted face-to-face. Four in-depth interviews were conducted online.

**Participants:**

Focus group discussions were conducted among twenty-three street food vendors, twenty-one caterers and seventy-six consumers of various eateries. In-depth interviews were conducted among two street food vendors and four caterers, individually.

**Results:**

Consumers and food operators perceived a high-salt intake within Malaysia’s out-of-home food sectors. Food operators emphasised the necessity for a comprehensive salt reduction policy in the out-of-home sector involving all stakeholders. Consumers faced limited awareness and knowledge, counterproductive practices among food operators and challenges in accessing affordable low-Na food products, whereas food operators faced the lack of standardised guidelines and effective enforcement mechanisms and uncooperative consumer practices. Both groups expressed that food quality and price of salt were also the barriers, and they advocated for awareness promotion, enhanced regulation of manufactured food products and stricter enforcement targeting vendors. Consumers also suggested promoting and recognising health-conscious food premises, whereas food operators suggested on knowledge enhancement tailored to them, strategies for gaining consumers acceptance and maintaining food quality.

**Conclusions:**

These findings provide valuable insights that serve as foundational evidence for developing and implementing salt reduction policies within Malaysia’s out-of-home sectors.

Urbanisation in Malaysia has provided new opportunities for Malaysians to eat out more frequently, driven by the expanding range of out-of-home food options^(1)^. In this context, eating out refers to the consumption of food prepared outside the home by various food vendors, such as in food courts, food stalls, *Mamak* stalls (Indian Muslim stalls), restaurants, western fast foods, food trucks and hawker stalls, including takeaway or delivery services for indoor, office or home consumption^(2)^. According to the Malaysian Adult Nutrition Survey (MANS 2014), 70 % of the Malaysian population regularly eat out, a particularly prominent trend among those residing in Peninsular Malaysia and urban areas^(3)^. In 2019, household expenditure on out-of-home foods among Malaysians increased to 11·2 % from 8·7 % in 2004/2005^(4)^. Consuming out-of-home food during various mealtimes, especially breakfast, has become popular among consumers in countries such as Uganda^(5)^ and Malaysia^(6)^.

In light of these circumstances, it is imperative to analyse the growing trend of eating out from a nutritional standpoint, considering the mounting evidence linking consumption of out-of-home foods to issues such as excess body weight^(7)^, obesity^(8)^ and increased risk of non-communicable diseases^(6)^. This alarming phenomenon is predominantly attributed to the high intake of fat, salt and calories in food^(5,7)^. The connection between excessive dietary salt intake and hypertension^(9–11)^ has become increasingly apparent. The 2019 National Health and Morbidity Survey underscored the persistently high prevalence of hypertension among adults in Malaysia at 30 %^(12)^. Malaysians consumed an average of 7·9 g of salt daily^(13)^, exceeding the maximum recommended limit of 5 g/d by the WHO^(14)^.

The recent National Strategy for Salt Reduction 2021–2025 steadfastly pursues the global target of achieving a 30 % reduction in salt or Na intake by 2025^(15)^ within the population. Based on the 2015–2020 strategy^(16)^, the current endeavour sets a long-term target of 6·0 g of daily salt intake^(15)^ by 2025. A midterm evaluation^(17)^ of the previous strategy revealed that salt levels of out-of-home foods have not been comprehensively monitored compared with packaged foods, although these foods are a common source of unhealthy food choices among Malaysians. As such, this outcome emerged as a notable barrier hindering salt reduction efforts in Malaysia, prompting the present strategy’s emphasis on monitoring the salt levels in such foods. Another study^(18)^ on Malaysians who frequently eat out revealed that the unavailability of healthy out-of-home food options as a prominent barrier impeding their adoption of healthier dietary habits. This issue is exacerbated among the low-income group in Malaysia, who often resort to unhealthy diets and frequent eating out^(19)^. In addition, a previous study^(20)^ demonstrated the feasibility of dietary modifications within this group, emphasising the significance of improving the out-of-home food landscape. Therefore, this study aimed to explore the perspectives, barriers and enablers of salt reduction within Malaysia’s out-of-home sectors, focusing on street food vendors, caterers and consumers. Accordingly, the findings of this study contributed essential insights for formulating an inclusive nutritional policy aimed at salt reduction within the out-of-home sector.

## Methods

This qualitative study design was adopted from a previously published primary research protocol^(21)^. This protocol employed focus group discussions (FGD) and in-depth interviews (IDI). The study adhered to the guidelines outlined in the Consolidated Criteria for Reporting Qualitative Research^(22)^ to ensure comprehensive reporting of qualitative research, encompassing all requisite details as specified for a qualitative study (see Appendix A).

### Participant recruitment and sampling

This study’s participants encompassed street food vendors, caterers and consumers from all five regions of Malaysia: West, North, South, East Coast regions of West Malaysia and east Malaysia. Street food vendors are defined as those who operates ready-to-eat food stalls for at least 2 years or more. Caterers consisted of individuals affiliated with reputable associations, including the Indian Muslim Restaurants Association, Chef Association, franchised food vendors, wedding caterers and school canteen operators. Consumers recruited through various eateries were those who habitually consumed outside meals at least three times a week. Consumers adhering to a low-salt diet or those dealing with conditions such as renal failure, heart failure or hypertension were excluded from this study^(21)^.

Participants were recruited through on-field approaches, established contacts and networking. Besides that, a roster of potential participants was compiled using diverse sources, including website searches, the Federal Agricultural Marketing Authority, and the Malaysian Agriculture Research and Development Institute. Invitations were extended through emails and telephone calls. As much as fifty street food vendors, fifty caterers and fifty consumers were invited. Those who were interested were required to complete an online registration form, providing their name, affiliation within the stakeholder group and contact information. Subsequently, these individuals were contacted to arrange the scheduling of their respective FGD sessions. Based on the snowball sampling technique, participants were encouraged to invite others who shared interest and availability to participate in the FGD session on the designated date. Individual IDI sessions were arranged for those unable to attend the FGD session. Table 1 shows the distribution of participants across the three stakeholder groups based on FGD and IDI sessions and regions. The response rate for this study was 50 % for vendors (*n* 25), 50 % for caterers (*n* 25) and 152 % for consumers (*n* 76).

**Table 1:** Distribution of participants across stakeholder groups by FGD/IDI and regions

Region	Focus group discussion (FGD)	In-depth interview (IDI)	Total response according to stakeholder groups
West	(FGD 1) 5 street food vendors	(IDI 1) 1 caterer	5 street food vendors
	(FGD 2) 7 caterers		8 caterers
	(FGD 3) 7 consumers		14 consumers
	(FGD 4) 7 consumers		
North	(FGD 1) 5 street food vendors	(IDI 1) 1 caterer*	5 street food vendors
	(FGD 2) 6 consumers	(IDI 2) 1 caterer*	2 caterers
	(FGD 3) 10 consumers		16 consumers
South	(FGD 1) 3 street food vendors	(IDI 1) 1 street food vendor*	5 street food vendors
	(FGD 2) 3 caterers	(IDI 2) 1 street food vendor*	3 caterers
	(FGD 3) 6 consumers		13 consumers
	(FGD 4) 7 consumers		
East coast	(FGD 1) 5 street food vendors		5 street food vendors
	(FGD 2) 3 caterers		3 caterers
	(FGD 3) 6 consumers		13 consumers
	(FGD 4) 7 consumers		
East Malaysia	(FGD 1) 5 street food vendors	(IDI 1) 2 caterers	5 street food vendors
	(FGD 2) 3 caterers		9 caterers
	(FGD 3) 4 caterers		20 consumers
	(FGD 4) 5 consumers		
	(FGD 5) 5 consumers		
	(FGD 6) 5 consumers		
	(FGD 7) 5 consumers		
Total	22 sessions (face-to-face)	2 sessions (face-to-face)	25 street food vendors
		4 sessions (online)	25 caterers
			76 consumers

* Session conducted online.

### Pilot study and instrument

The questionnaires utilised for the FGD and IDI were developed based on a combination of the social ecological model^(23)^, with adaptations derived from the UK Medical Research Council Guidance on Evaluating Complex Intervention^(24)^ and the Theoretical Domains Framework^(25)^, as described in the study protocol^(21)^. A pilot study was conducted among five individual representatives from the targeted participant pool in the central region to validate the questionnaire. Subsequently, refinements were implemented to enhance questionnaire clarity and relevance. This study employed distinct sets of questionnaires for street food vendors and caterers and consumers (see Appendix B). Before commencing the first session of FGD and IDI sessions, all research team members (comprising seven females, two males) underwent comprehensive training, including simulated sessions. This training familiarised the team with their roles as interviewers or moderators, honing their probing skills for addressing specific points of interest, cultivating prompting technique and other appropriate skills.

### Data collection

The data collection process spanned from March 2020 to May 2022. A total of twenty-two FGD and two IDI were conducted face-to-face and situated in comfortable meeting rooms. Meanwhile, four IDI were conducted online through Google Meet due to COVID-19 restrictions. Each session comprised the participant/s, a researcher as the moderator or interviewer, and additional researcher tasked as a note-taker. The note-taker’s role entailed transcribing noteworthy topics discussed during the session, aiding the subsequent data analysis process. Before the commencement of each session, the roles of moderator/interviewer and note-taker were clarified with the participants, accompanied by a brief overview of the study’s purpose. Participants were informed about the audio recording of the sessions. They were also given an information sheet and a consent form in Bahasa Malaysia and English language. Once the completed consent forms were returned, the sessions commenced. The face-to-face FGD and IDI sessions ranged from 1 hour to 2 hours, and these interactions were documented with a digital voice recorder. In contrast, online IDI sessions lasting between 45 min to 1 hour were recorded using Google Meet. At the conclusion of each session, socio-demographic data were collected.

### Data management

The audio recordings of the IDI and FGD sessions were transcribed verbatim. Throughout the transcription process and subsequent data reporting, each participant was allocated a pseudonym comprising their stakeholder group affiliation, a coded number, geographical zone and gender. This practice was adopted to maintain the confidentiality of all participants involved in the study.

### Data analysis

The transcripts underwent analysis using an inductive thematic approach facilitated by NVivo (version 12; QSR International, Doncaster). The transcripts were initially assigned open codes during the analysis process, which were subsequently refined and amalgamated to form conceptual codes and sub-codes. This iterative process involved two researchers collaborating to establish a consistent coding framework, which was then subject to discussion and consensus within the research team. Codes that had been refined and shared similar connotations were either grouped into sub-themes or retained as standalone concepts. The emerging themes were then developed and discussed by the research team. These themes, accompanied by their corresponding sub-themes, were subsequently categorised into perceptions, barriers or enablers. To provide the descriptive insights into the participants’ socio-demographic characteristics, they were analysed descriptively using Statistical Products and Service Solution (SPSS) (version 25; Inc.).

## Results

### Socio-demographic characteristics of the participants

FGD and IDI were conducted across three distinct stakeholder groups: consumers of out-of-home foods (*n* 76), street food vendors (*n* 25) and caterers (*n* 25). Table 2 summarises the participants’ socio-demographic data in the three groups. Overall, the median age of participants in this study was 36 years. Predominantly, the participants were women (51·6 %) and had attained a tertiary level of education (61·9 %). The median and mean age were 38 and 39 years among street food vendors and caterers, respectively. Most vendors (60 %) and caterers (60 %) were men, and their educational qualifications varied between secondary (48·0 %) and tertiary (72·0 %) levels. On the other hand, consumers had an average age of approximately 35 years, with a higher representation of women (59·2 %). Most individuals within this category had achieved a tertiary education level (61·9 %).

**Table 2:** Sociodemographic data of the participants

Sociodemographic information	Stakeholder group						Total participants (*n* 126)	
	Food operator							
	Street food vendors (*n* 25)		Caterers (*n* 25)		Consumers (*n* 76)			
Age (years)								
Median	38·0		–		35·0		36·0	
IQR	12·0		–		11·5		13·0	
Mean	–		38·8		–		–	
s d	–		11·0		–		–	
Range	24–73		17–65		24–62		17–73	
Gender	*n*	%	*n*	%	*n*	%	*n*	%
Men	15	60·0	15	60·0	31	40·8	61	48·4
Women	10	40·0	10	40·0	45	59·2	65	51·6
Education level								
Primary	0	0·0	1	4·0	0	0	1	0·8
Secondary	12	48·0	4	16·0	18	23·7	34	27·0
Tertiary	11	44·0	18	72·0	49	64·5	78	61·9
No education	2	8·0	2	8·0	0	0	4	3·2
Not available	0	0·0	0	0·0	9	11·8	9	7·1

Table 3 presents an overview of themes and sub-themes related to the consumers’ perceptions, barriers faced and identified enablers concerning salt reduction within Malaysia’s out-of-home sectors. Further, Table 4 outlines the prevailing perceptions, encountered barriers and recognised enablers regarding salt reduction within Malaysia’s out-of-home sectors, specifically among the food operator group encompassing street food vendors and caterers.

**Table 3:** Perceptions, barriers and enablers regarding salt reduction in Malaysia’s out-of-home sectors identified by consumers

Consumers perceptions regarding salt intake in Malaysia’s out-of-home sectors	
Theme/sub-theme	Example of quotes
Salt intake in Malaysia	
High-salt intake and health complications	‘I also think that salt intake among Malaysians is high. We can’t even control how much salt we consume in our daily lives.’ (C6/S/Male) ‘..associated with heart disease… Hypertension, heart disease, blockage…’ (C2/N/Male)
Factors influencing salt intake in out-of-home food	
Various sources of high-salt food	‘…so, whether we eat at home or outside we tend to take processed foods which are salty…’ (C2/W/Male) ‘…I admit that I rather like my food a bit salty and sometimes we eat junk foods which actually are salty…’ (C4/EC/Female) ‘Fast food, because it has a lot of salt…’ (C3/N/Male)
Convenience of out-of-home food options	‘Usually when working, during lunch, I have to eat outside. When coming off from work, it is tiring, so I have to eat outside too.’ (C3/EM/Male) ‘It’s easy to order. There are many delivery services nowadays. So it’s easy to order (C2/EM/Male) and thus making harder to control salt intake daily…’ (C6/W/Female) ‘For me, the circumstances force me to eat outside. I have no one to cook at home. So, I have no other options but to eat outside. Similar to what C3 said previously, I am the type to hang out and eat.’ (C2/EM/Male)
Incorporation of salt, seasonings and sauces by food operators	‘…if we eating outside, when they cook fried rice, they will put in maggi (seasoning), ajinomoto (seasoning), salt and also soy sauce and whatnot.’. (C2/EC/Female) ‘…lots of MSG is added into the keropok lekor (fish sausage). Food bought from night market, RM 1 fried chicken also salty…’(C2/EC/Female) ‘Foods that are sold at side roads. Fish sauce. and also lots of MSG.’(C1/EC/Female)
Consumers’ out-of-home food practices	‘First, if I go to restaurants or any eateries, I choose food that has less salt. Or if not, we demand, request for noodles - noodles with less salt. Such as less salt mi kolok.’ (C7/EM/Male) ‘Yeah, if I buy food from mamak (Indian Muslim Restaurant), I will alter the food back when at home. I add more water into it…” (C6/W/Female) … if it tastes salty, I will adjust the taste when at home.’ (C5/W/Female) ‘The first time eating less salty fried rice. It was definitely not salty. I needed to add salt into it.’ (C1/EM/Male)
The practice of reading nutritional content labels on packaged foods among consumers	‘We look at products that we use every day. Did not look at the nutrition content. We only look at the price. That is the problem. This product is expensive, this product is not.’ (C6/W/Female) ‘Ingredients is the most important thing.’ (C3/EM/Male) ‘Meaning that I do not care that much on the nutritional content. What is important is the halal logo and MeSTI logo, that’s all.’ (C1/EM/Male) ‘Usually, I inspect the expiry date. For salt, I do not focus on it that much.’ (C5/W/Female) ‘Probably because we do not understand much on the nutrition content label. So, I do not look at it that much.’ (C3/EC/Female)
Barriers encountered by consumers regarding salt reduction in Malaysia’s out-of-home sectors	
Theme/sub-theme	Example of quotes
Awareness and knowledge among consumers	
Inefficient salt-related messaging	‘Yes, felt that the messages did not really deliver to us. The messages did not have a wide coverage compared to sugar messages…so I do not see why I should eat less salt when eat home or outside.’ C5/W/Female)
Challenges from food operators’ practices	
Limited knowledge and awareness to reduce salt in food	‘…sometimes we already tell them to use less salt, don’t make it salty. But, it was still salty.’ (C4/N/Female) ‘…yes it is probably because they do not care…or don’t know how to (use less salt).’ (C5/N/Female)
Food operator autonomy in food preparation	‘Sometimes it’s because we are not the cook, so when the cook tends to cook salty food, we as customers just eat it…’ (C1/N/Female) ‘Regarding salt usage, it depends on the cook, we just eat whatever they cook…’ (C4/N/Female)
Food quality	
Effect on taste, flavour and mouthfeel of reduced-salt foods and beverages	‘It is hard not to put salt in certain dishes, because sometimes the salt enhances the taste.’ (C4/EC/Female) ‘If salt is not added, the creaminess and mouthfeel of the food won’t be enhanced.’ (C7/EM/Male)
Price of regular salt	‘…for eating, sometimes people are more concern on the cost rather than the nutrient contents. And…salt is a cheap thing…’ (C2/EC/Female) ‘The salt reduction campaign…salt itself is not that pricey.’ (C1/EC/Male)
Availability and affordability of low and high-salt food products in the market	
Lack of low-salt food products	‘I think low salt products are not that many in the market. Even if there are a few, probably we didn’t notice that.’ (C3/EM/Male) ‘Low salt food products are really only a few that we can see. Usually, we would see low sugar products, not the low salt ones.’ (C1/EM/Female) ‘No, I don’t think I have seen low salt products in the market. Are there any?’ (C1/W/Female); (C2/W/Female); (C5/W/Female); (C6/W/Female)
Price of low-salt food products	‘Because the healthier versions are always pricier than the regular versions.’ (C1/W/Female)
Enablers identified by consumers regarding salt reduction in Malaysia’s out-of-home sectors	
Theme/sub-theme	Example of quotes
Raising awareness among consumers	
Promoting health as the ultimate goal	‘Means that I can see our enthusiasm to take care of our health. So this campaign is good so that we can be given good health.’ (C1/EM/Male) ‘To me, I am willing to join in the campaign because I want prevent high blood pressure.’ (C2/EM/Male)
Dissemination of knowledge on salt reduction	‘Have to educate through self-awareness. For example, advertise the health effects like high blood pressure. That is one effective way to do it.’ (C2/EM/Male) ‘Maybe the media can teach people how to read labels, salt content, something like that.’ (C4/W/Female) ‘Then have to inform on the salt substitute.’ (C6/W/Female) ‘So that Malaysians know how to read label. Then, they will also know how and how much salt that can be taken daily and used in cooking.’ (C5/W/Female)
Effective communication channels	‘Except for the way to deliver…(C2/EC/Female) The delivery needs to be in a way that is easy to be understood.’ (C1/EC/Female) ‘We can disseminate through Facebook, WhatsApp, Instagram and many more. I think if it’s frequently played in mass media, probably the society can accept it. Like the case of covid now.’ (C5/W/Female) ‘But maybe for salt usage, maybe posters on heart attacks and all. Can be displayed to everyone. So that they can straight away see that high usage of salt can cause heart attack etc.’ (C1/EM/Male) ‘Or someone giving talks to the crowds.’ (C4/W/Female) ‘Programs like food selling. Because if there are food festivals, maybe we will be more interested.’ (C2/EC/Female)
Targeting younger consumers	‘For me, I think there need to be an exposure from school. Lots of campaigns on salt in food. That is one way of it.’ (C2/EM/Male) ‘Can do those kinds of programs, like awareness campaign. If it can be start early, through the school curriculum, that would be good. MOH can visit schools and do a session with the Education Division of every area.’ (C3/EM/Female) ‘To me, need early education. For example, in primary schools, high schools, kindergartens…we inform the children about salt. So, they can tell their parents when at home like “Mom, this food is salty. The teacher said that it is not good,” for example.’ (C2/N/Female)
Promotion and recognition of health-conscious food premises	‘Maybe the government can organise a program that is specifically for the stalls that take care of their salt usage in each of the food. So, if they do it, probably through advertisements, we could know that this and that stall is good, healthy. So, maybe we as customers will become interested to visit the stall.’ (C2/S/Female) ‘One program that can introduce those kinds of stalls.’ (C1/S/Female) ‘The government and vendors can promote their food and healthy stalls…Or the health ministry can issue a certification for the healthy stalls…’ (C2/N/Female); (C4/N/Male)
Regulation measures for manufactured food products	
Control over regular salt distribution	‘But the government doesn’t do any control (on the salt content in food products). There have to be an explanation, or an act that controls the sales of salt…’ (C7/EM/Male) ‘Please reduce the selling of salt in the market. Then there would not be too many salt manufacturers anymore.’ (C4/EM/Male)
Enforcement of mandatory salt limits	‘Next, for the industry, there must be an enforcement. We legislate law to limit the salt usage in the food. That’s one way of it.’ (C2/EM/Male) ‘To me, for food industries and manufacturers, we can set the requirements, such as the percentage of salt that is allowed in the products. So, they will proceed to produce products that follow the requirement.’ (C2/N/Female) ‘Set a maximum limit for each product. To limit the salt usage.’ (C10/N/Male)
Integration of salt substitutes in food products	‘Because if we want to reduce or use no salt at all for pickled products and other high salt products, then what will happen to the manufacturers? So, the government must have a product that can substitute salt. Just like how stevia can replace sugar. Because previously for sugar campaign, it’s because we already have the substitute for it. So, what is the alternative for salt?’ (C9/EM/Female)
Enhancement of food packaging labels	‘That is important as well (to put a warning sign on the salt packaging).’ (C2/S/Female) ‘Yes, in terms of the nutrition information.’ (C7/N/Female) ‘…I suggest that the products in the market to be put some colours on it. For example, put red for salted fish that has the most salt. Maybe we can put green for the one with lowest salt. The colour system can help even the people who can’t read. Like “Oh, this one is red, so this must have been the highest in salt.’ (C7/EM/Male)
Affordability of low-salt food products	‘We will choose the ones that are cheaper (C6/W/Female) …right, because the healthier versions are always pricier than the regular versions.’ (C1/W/Female) ‘Reducing the price (of the food products) can attract Malaysians to buy. When it became cheap, we will buy it. We will support.’ (C5/W/Female)
Price adjustment and taxation of regular salt	‘Increase the price of salt.’ (C2/EM/Male); (C8/EM/Female) ‘Maybe taxation should be introduced to the industries as well.’ (C9/N/Male) ‘Due to everyone use salt, maybe the taxation needs to be a mandatory.’ (C5/N/Male)
Regulation of street food vendors	
Monitoring and enforcement of salt content	‘Maybe the health division can do monitoring on the stalls to limit the salt usage in every food. If there are stalls or restaurants that use a lot of salt, maybe MOH or other agency can give exposure to them on reducing the salt in food.’ (C1/S/Female)

*C, Consumer; W, West; N, North; S, South; EC, East Coast; EM, East Malaysia.

**Table 4:** Perceptions, barriers and enablers regarding salt intake and salt reduction in Malaysia’s out-of-home sectors identified by food operators

Food operators perceptions regarding salt intake and salt reduction in Malaysia’s out-of-home sectors	
Theme/sub-theme	Example of quotes
Salt intake in Malaysia	
High-salt intake and health complications	‘In my view, the salt intake in Malaysia is quite high. Because if we look at the nutrition aspect, we can know that the salt intake is high.’ (CO1/EM/Male); (SFV16/W/Male)
Factors influencing salt intake in out-of-home food	
Various sources of high-salt food	‘Sometimes there are food that are added with salt, sugar, and monosodium glutamate to get viral. But try sending the food for analysis, the salt must be high.’ (CO1/W/Male) ‘Salt intake is high especially traditional food that definitely uses a lot of salt.’ (SFV5/EC/Male) ‘…as you can see, shrimp paste, anchovies are high in salt content…’ (C7/C/Male)
Incorporation of salt, seasonings and sauces by food operators	‘Yes, I do add a little bit of seasonings because that gives the flavour.’ (SFV19/EM/Female)…some vendors replace salt with seasonings.’ (SFV9/N/Male) ‘…when we cook, we put in multiple sources of salt.’ (SFV18/EM/Male) ‘Like I said just now, chicken stock, monosodium glutamate, chicken broth. We use all these.’ (Caterer 3/West/Male) ‘Yes, I add salt into our mango float menu in order to enhance its taste.’ (SFV14/W/Male) ‘Like milk tea, they (the vendors) will add salt into it to add the creaminess.’ (SFV7/N/Female) ‘There are some people that buy soft drinks and ask to add in salt.’ (CO5/W/Male)
The practice of reading nutritional content labels on packaged foods among food operators	‘We only take the products that we usually use. If we compare with other brands, we usually compare the price, halal logo, MeSTI logo, and ingredients. Not really on the nutrition label.’ (SFV21/EM/Male) ‘Have never looked at the nutrition label.’ (Caterer 5/East Malaysia/Female)
Salt reduction policy	
Inclusive engagement	‘To reduce salt, I think all parties should be involved in the policy… the food providers, consumers, government… (CO1/W/Male)… all parties, then the citizens will support as well.’ (SFV4/EC/Male) ‘The food manufacturers for high salt processed food should take part in the policy.’ (SFV6/N/Male) ‘…salt usage among vendors is not that extreme compared to the big industries. Big industries use a lot of salt especially in fast food and in junk food. These contribute to the high salt intake compared to the small vendors that sell traditional food.’ (SFV4/EC/Male)
Policy implementation approaches	‘To start, the policy should be on a voluntary basis first. Maybe for the first two years.’ (SFV18/W/Male) ‘…volunteer first…’ (CO7/W/Male) ‘The policy should be done voluntarily. If it is done mandatory, it will be like the smoking campaign. People still smoke.’ (SFV13/S/Female) ‘For me, it needs to be mandatory. Maybe need to have a policy.’ (CO3/S/Male) ‘…when it is not mandatory, like viral food, supposed the salt cannot be high. So, need to have a law and regulations.’ (CO1/W/Male)
Barriers encountered by food operators regarding salt reduction in Malaysia’s out-of-home sectors	
Theme/sub-theme	Example of quotes
Lack of a standard guideline	‘Yes, so far for catering, there is not standard operating procedure for the food preparation. So, we just cook accordingly.’ (CO1/EM/M) ‘Just like we said earlier, we don’t see the guideline to reduce salt usage.’ (SFV4/EC/Male) ‘In terms of awareness, they do not know the ingredients that they can use to substitute the salt or reduce the salt.’ (SFV14/W/Male) ‘Because they do not know how to change the formulation of their recipes and still taste good.’ (Street Vendor 24/South/Male)
Challenges from consumer practices	
Awareness of the importance of salt reduction	To me the most important thing is knowledge. How we are going to tell the society or customers that this salt reduction is very important. That for me is a challenge…” (CO3/S/Male) ‘To be honest, as far as I know, I have never meet customer that request less salt for their dishes. It’s because they don’t have the awareness.’ (CO1/W/Male) ‘The parents sometimes asked for salty soy sauce for their kids at our restaurants. They insisted on it even after I said that the food is already salty. So, this habit is a problem…’ (CO3/C/M).
Consumer acceptance	‘Usually, my customers will complain if my food is not salty.’ (SFV7/N/Female) ‘They definitely will complain that the food tasted bland…not tasty.’ (CO5/W/Male) ‘If we change the formulation of the recipes, customers might not like it.’ (SFV24/S/Male) ‘We cannot sell food that is cooked according to our preference. It must follow the preference of our customers.’ (SFV19/EM/Female) ‘…maybe the number of customers will reduce, they will say it’s better for them took at home rather than buying from us.’ (CO1/EM/Male)
Lack of consumers request for reduced salt	‘To be honest, as far as I know, I have never meet customer that request less salt for their dishes.’ (CO1/W/Male) ‘My customers don’t really ask for less salt.’ (SFV7/N/Female)
Food quality	
Effect on taste, flavour and mouthfeel of reduced-salt foods and beverages	‘Cannot be reduced (salt) as it will give effects to the taste and quality of the food.’ (SFV5/EC/Male) ‘Usually, we add salt into creamy dishes to enhance the flavour and creaminess. So if we reduce the salt, it would not be creamy or tasty enough for the consumers.’ (SFV25/S/Female)
Effect on the taste of traditional or heritage recipes	‘It’s not easy to change recipes for traditional food because that is where the “tradition” sits. If customers eat, then they will complain that it doesn’t taste like how it should.’ (SFV5/EC/Male) ‘It’s because these recipes are passed down from our ancestors, so we are used to follow the recipes.’ (SFV6/N/Male)
Price of regular salt	
Comparison of regular salt prices with natural flavour enhancers and salt substitutes	‘If the cost is too expensive, I think vendors would not use KCl.’ (SFV24/S/Male) ‘…but for cost wise, we cannot replace the cheap salt with all these wet spices in high volume. Lemongrass, bird’s eye chili is RM 15 per kg.’ (CO1/W/Male)
Regulation of street food vendors	
Lack of regulatory authority	‘For vendors, they do not have any regulations or rules to follow to prepare healthy food, except for cleanliness and license. So, they prepare the food in their own way because they do not have any regulatory body.’ (SFV24/S/Male)
The tedious license registration process	‘We would not attend the course (preparing less salt food) because most of us do not have license. So I think only those who have license will attend the course.’ (SFV/21/EM/Male) ‘It’s like this, the procedure to get the license is too long and complicated… so, that is why I don’t have the license yet.’ (SFA/20/EM/Male)
Enablers identified by food operators regarding salt reduction in Malaysia’s out-of-home sectors	
Theme/sub-theme	Example of quotes
Enhancing knowledge among food operators	
Establishing a standard guideline for salt reduction in food	‘To me, the first thing is the guideline. What I mean is, how much salt that we are supposed to use. The small vendors want to know that.’ (SFV4/EC/Male) ‘I think for caterers, their own standard operating procedures need to be made first.’ (CO1/EM/Female)
Implementation of mandatory courses or trainings	‘How can we practice using less salt in food? Maybe through classes conducted by the authorities…’ (SFV5/EC/Male) ‘So maybe, part of the food handling courses also, can also talk about eating healthy. Not only clean in the kitchen…’ (CO3/N/Male) ‘Because we already have the food preparation course right, so just include this one in this course.’ (CO1/EC/Female) ‘We try to make it (attending healthy food preparation course) as a mandatory for those that want to get business licence from the city council, they need to attend the healthy food preparation course.’ (CO1/W/Male)
Leveraging guidance from research agencies	‘We can ask support from MARDI to do our R&D such as how to cook the food without salt or with less salt, and then can also be applied to ready-to-eat food.’ (SFV5/EC/Male) ‘How much to reduce… based on the standardised recipe. Let say in the recipe, stated 100 g and we reduce to 50 g. So, what is the difference? That means we need a professional to do the research.’ (CO2/W/Male)
Raising awareness among consumers and food operators	
Promoting health and managing healthcare expenses as the ultimate goals	‘I would join in the policy if it’s for the health of the public.’ (SFV9/N/Male) ‘We support the salt reduction for our health.’ (CO1/W/Male) ‘I think this is let it become, actually this is a very good calling, these calling, it is a hard calling, you know not only that the action very root, the effective will be helping people, reduce the medical spending, and always saving money.’ (CO7/W/Male) ‘I have high blood pressure, so it costs so much every time I have to get medications even from government hospitals. So that is why I join this discussion session.’ (SFV18/W/Male)
Educating consumers on the importance and methods of salt reduction in food	‘Creating awareness among the consumers is important. Creating awareness among the food sectors is also important.’ (C1/W/Male) ‘We need awareness among the customers as well.’ (SFV13/S/Female) ‘Yes, customers’ request is the most important thing.’ (SFV19/EM/Female) ‘We follow the request from customers when preparing food.’ (SFV13/S/Female) ‘Other than that, for things like how to eat balance, diagram of food pyramid, MOH can turn that into visual and make them in a form of sticker or any printing materials. Like us, we have trucks or stalls, we can paste them everywhere, so people can read when they are waiting for their order.’ (SFV17W/Female)
Targeting younger consumers	‘Another thing is, we must educate on salt intake and salt in food starting from school level. We could design a subject especially for it.’ (SFV18/W/Male) ‘For me, school is the most effective. Teachers need to play their vital role because they can teach a culture. Less salt food culture. The students need to be given awareness to avoid instant food.’ (CO1/EC/Female)
Strategies for gaining consumers acceptance	
Progressive salt reduction and separate salt presentation	‘I think it depends on customer, if the food providers sell less salty food for example the salty fish is not salt anymore, it will ruin the business because customer want their dish to be delicious. When you implement drastically, the reputation suddenly…’ (CO2/W/Male) ‘They do not have to mention. If we want to reduce, we just reduce.’ (CO4/W/Male) ‘We can prepare salt on the table, and the customers can add salt themselves.’ (SFV6/N/Male)
Introduction of reduced-salt menus	‘Those that come to buy can have a taste of the reduced-salt food. They can have a new kind of flavour.’ (SFV12/S/Male) ‘As you can say, the benefit is that you might get additional customer. You might lose a few, but you can still have new customer that is keen in eating healthy. So, you will not lose them. I don’t think you will lose 100 % of them.’ (CO3/N/Male) ‘We can do food festival consisting of programs with vendors demonstrating their food using original recipes and reduced-salt food.’ (SFV2/EC/Male); (SFV17/W/Female)
Promotion and recognition of health-conscious food premises	‘…for example, we give a recognition to hotels or restaurants through invoice prove. Let say last month they bought 3 kg but this month 2 kg, so we give them the recognition…if we put rating like 5-star rating for example, will catch their attention. This is their perception.’ (CO1/W/Male)
Food quality	
Salt reduction through the freezing method	‘When the product is in a frozen form, customers will be fine with it. I have tried with my traditional food menu.’ (SFV5/EC/Male) ‘Salt can be reduced in traditional food, but it needs to be frozen because the customers will already expect that the food will not have 100 % of its original taste. So, from that aspect, we could reduce the salt usage. But cannot for ready-to-eat food.’ (SFV5/EC/Male)
Salt reduction in spices-infused dishes	‘I think dishes like curries are suitable if we want to add more spices and lower the salt.’ (SFV19/EM/Female) ‘We got our own traditional style (for Indian Muslim dishes). So salt is not so important in our cooking. If we put lots of onions, it got all the taste. Normally spices and onions. The onions enhance the taste.’ (CO6/W/Male) ‘In Indian foods…when we put all these wet spices, we fry them well, we cook them well, sometimes we do not need salt. That has been implemented in the industry.’ (CO1/West/Male) ‘I only use spices for my burger patties. And maybe the smoke already gives the flavour to the patties, so I do not add salt to it.’ (SFV2/EC/Male)
Regulation measures for manufactured food products	
Enhancement of food packaging labels	‘The picture on the food packaging. Let say, if lots of salt, maybe can show maggots or stomach cancer, high blood pressure.’ (CO3/W/Male)
Control over regular salt and high-salt products distribution	‘…There are lots of salt, lots of sauces. So, if the display is reduced. They will finally disappear from the industry.’ (CO5/W/Male)
Price adjustment and taxation of regular salt	‘I suggest we increase the salt price.’ (CO1/W/Male) ‘Tax would be helpful. It will help us in controlling the salt usage.’ (CO2/EM/Male) ‘Imposing taxation would be a great suggestion.’ (CO/EM/Male)
Price adjustment of natural flavour enhancers and salt substitute	‘…for me, maybe can reduce the price of herbs, spices.’ (CO3/S/Male) ‘Like I said, pricing point of KCl is very important. If the cost is too expensive, I think vendors would not use KCl.’ (SFV 24/S/Male) ‘Yes, most importantly, the government should take care of the price of KCl first, then there would not be any problem for the industry players to accept the salt reduction initiative.’ (SFV24/S/Male)
Regulation of street food vendors	
Collaborative partnership between MOH and local authorities	‘Now, vendors are only tied to the local authorities for their business license. I think MOH can collaborate with them to extend the information on the salt reduction policy…including making sure they use less salt in food.’ (SFV24/S/Male) ‘Yes, the local authority need to make the procedure easier for us to get our license.’ (SFV24/S/Male)

*SFV, Street Food Vendor; CO, Catering Operator; W, West; N, North; S, South; EC, East Coast; EM, East Malaysia.

### Consumers perceptions regarding salt intake in Malaysia’s out-of-home sectors

#### Salt intake in Malaysia

##### High-salt intake and health complications

The prevalent sentiment among consumers is the recognition of high-salt intake levels in Malaysia, which potentially contributes to health issues. Most consumers acknowledged the pervasive consumption of salt among Malaysians and the adverse health effects, such as hypertension and heart disease.

#### Factors influencing salt intake in out-of-home food

##### Various sources of high-salt food

Consumers identified diverse high-salt food sources, encompassing processed, junk and fast food. The accessibility and prevalence of these options contributed to the high-salt intake within Malaysia. Such foods were often consumed at out-of-home locations or brought home.

##### Convenience of out-of-home food options

Consumers leading busy lives, particularly those with work commitments, expressed that out-of-home food are convenient, especially for lunch and dinner. This sentiment was predominant among consumers from East Malaysia and the West region, who emphasised increasing access to out-of-home food options through food delivery services. Similarly, individuals living alone without a penchant for cooking appreciated the social aspects of eating out, such as the opportunity to meet and mingle with friends and family. Conversely, some consumers who usually cook at home found solace in occasionally eating out to escape the kitchen, also noting cost-effectiveness as a factor.

##### Incorporation of salt, seasonings and sauces by food operators

Some consumers mentioned that food operators often incorporate seasonings, salt and sauces into food sold in restaurants, roadside stalls and nighttime venues. This practice posed a challenge to managing salt intake when eating out.

##### Consumers’ out-of-home food practices

Consumers exhibited various behaviours when eating out that influenced their salt consumption habits. One consumer from East Malaysia highlighted the practice of selecting lower-Na options from the menu. In cases where such options were unavailable, they requested chefs to reduce the salt content in their meals. Similarly, some West Malaysian consumers took personal initiative by adjusting the saltiness of purchased foods once at home. However, among consumers from East Malaysia, there were instances where additional salt was added to purchase food if it is needed to be seasoned to their preference.

##### The practice of reading nutritional content labels on packaged foods among consumers

Majority of the consumers admitted that they did not have the habit of inspecting the nutritional content label when surveying food products in the market. Instead, they focused more on the price label, ingredients, halal certification, ‘Food Safety is the Responsibility of the Industry’ (MeSTI) label, and expiry date of the products. Some consumers expressed reluctance to scrutinise nutrition labels, citing challenges in comprehending the information provided.

### Barriers encountered by consumers regarding salt reduction in Malaysia’s out-of-home sectors

#### Consumer awareness and knowledge

##### Inefficient salt-related messaging

In discussions about salt reduction campaigns and messages promoted by the Ministry of Health (MOH), many consumers expressed dissatisfaction with the delivery of these messages. Consumers found that salt reduction campaigns needed more visibility and impact than other messages related to sugar and energy intake. Therefore, the significance of reducing salt intake in both home-cooked meals and out-of-home dining failed to resonate with them.

#### Challenges from food operators’ practices

##### Limited knowledge and awareness to reduce salt in food

Some consumers noted that their requests for reduced salt in their meals often needed to be met, as the food still tasted overly salty. This circumstance may be due to limited awareness and knowledge among food operators regarding the importance and methods of salt reduction.

##### Food operator autonomy in food preparation

A recurring challenge, as expressed by most consumers, was their limited ability to regulate salt intake when consuming out-of-home food. This challenge arose from the fact that the final salt content of the dishes was solely determined by the cooks, influenced by their personal preferences and palate. Consequently, consumers needed more control over the amount of salt in their chosen dishes.

#### Food quality

##### Effect on taste, flavour and mouthfeel of reduced-salt foods and beverages

All consumers agreed that salt enhances the flavour and taste of food. As a result, reducing salt content was perceived as compromising the overall taste and appeal of dishes. Beyond flavour enhancement, salt was recognised for enriching the creamy texture of specific foods and beverages. Notably, creamy food and beverages containing coconut milk and dairy were identified as general types of food with salt-induced creaminess and enhanced enjoyment in consumption.

#### Price of regular salt

A consumer stated that due to the low price of regular salt, there might be little concern among out-of-home sectors about reducing its usage.

#### Availability and affordability of low- and high-salt food products in the market

##### Lack of low-salt food products

Many consumers noted the market’s limited presence of low-salt food products in the market. They further observed that the healthier variants of commonly available products mainly focus on reducing sugar content.

##### Price of low-salt food products

Most consumers agreed that low-salt products generally have a higher price tag than regular salt products. As a result, this discourages the consumers and even food operators from incorporating low-salt options.

### Enablers identified by consumers regarding salt reduction in Malaysia’s out-of-home sectors

#### Raising awareness among consumers

##### Promoting health as the ultimate goal

Consumers exhibit a genuine interest in safeguarding their overall well-being. Therefore, focusing on health benefits as the campaign’s primary objective serves as a constant reminder to consumers about the advantages of reducing salt intake. This primary objective should be integrated into policies and campaigns.

##### Dissemination of knowledge on salt reduction

Consumers from East Malaysia suggested implementing a comprehensive awareness campaign to educate individuals about the vital significance of reducing salt in their diet. Educational messages should encompass instructive content, such as interpreting nutrition labels, understanding the role of salt substitutes, recognising diverse commercial salt varieties and their benefits and comprehending recommended salt allowances for consumption and cooking. In addition, outlining the health consequences of excessive salt consumption should be a focal point.

##### Effective communication channels

Consumers emphasised the importance of utilising appropriate communication channels to convey salt-related messages. One consumer noted that the messages would be more likely to be accepted if they were consistently promoted through social media. In addition to social media, visual aids, such as posters, banners and billboards, were also recommended by the participants. These visual mediums facilitate easy comprehension of the information. Verbal communication methods, including talks and presentations, were a means to reach larger audiences, particularly within community residences. Leveraging television and radio for advertisements between segments was also highlighted. Further, consumers were interested in participating in food festivals that feature reduced-salt food options.

##### Targeting younger consumers

Most consumers proposed initiating salt-related campaigns within educational settings, particularly schools, through collaboration with the MOH and Ministry of Education. This campaign approach involves all education levels, including kindergarten, primary and secondary schools. The participants emphasised that through such efforts, children can transmit the knowledge and awareness gained in schools to their families at home.

#### Promotion and recognition of health-conscious food premises

Some consumers proposed a government-led initiative highlighting food establishments specialising in low-salt food preparation. This programme would facilitate consumer identification of these establishments through advertisements or certifications.

#### Regulation measures for manufactured food products

##### Control over regular salt distribution

Consumers emphasised the necessity for governmental intervention to regulate the sales of regular salt within the market. Such regulation would result in a reduction in the number of salt manufacturers operating in the market.

##### Enforcement of mandatory salt limits

Consumers recommended the establishment of definitive salt content limits applicable to commercially produced food items. All food manufacturers must adhere to these limits, consequently steering the production of items within the prescribed salt limits. This measure would foster a market that predominantly offers low-salt food products.

##### Integration of salt substitutes in food products

Consumers recommended introducing alternatives to conventional salt for food manufacturers to facilitate the reduction of salt content, particularly in high-salt products. Encouraging the utilisation of salt substitutes would be essential in altering food product formulations, similar to how stevia is employed to replace sugar in sugar products.

##### Enhancement of food packaging labels

Consumers stressed the importance of refining the packaging of high-salt food products. The enhancement of food packaging labels includes warning labels of the adverse health conditions of excessive salt consumption and incorporating Na content details in the nutrition facts panel. To distinguish between low-, moderate- and high-salt food products, the consumers suggested implementing a traffic light labelling system to indicate the salt content levels using colours, aiding consumers in making informed choices.

##### Affordability of low-salt food products

Consumers indicated their willingness to purchase low-salt alternatives of commercially available food products if these options were reasonably priced. They acknowledged the prevailing trend where healthier alternatives tend to be priced higher than regular salt food products.

##### Price adjustment and taxation of regular salt

Based on input from consumers in the North, it was suggested that the price of regular salt should be increased along with the imposition of a salt tax. This dual approach discourages salt consumption within out-of-home sectors by making regular salt less economically viable.

#### Regulation of street food vendors

##### Monitoring and enforcement of salt content

A consumer suggested that MOH should monitor the salt usage and content on the menus of food stalls. This approach would involve transparently highlighting the efforts toward salt reduction in food offerings.

### Food operators’ perceptions regarding salt intake and salt reduction in Malaysia’s out-of-home sectors

#### Salt intake in Malaysia

##### High-salt intake and health complications

Most caterers and food vendors agreed that Malaysians consumed much salt, with a collective awareness that excessive salt intake could lead to health complications, such as hypertension and heart disease.

#### Factors influencing salt intake in out-of-home food

##### Various sources of high-salt food

Food operators highlighted several factors contributing to Malaysians’ salt intake patterns. These factors include the prevalence of high-Na food sources, such as street food, fermented dishes, staple Malaysian dishes and traditional food. Street food operators tend to add salt and monosodium glutamate, rendering the food Na rich. Fermented delicacies, such as shrimp paste, *budu* (a condiment made from anchovies) and salted fish, were recognised as additional sources of high-salt content. Staple Malaysian dishes, such as *asam pedas* (a dish comprised of fish cooked in a sour and spicy gravy), fried noodles, noodle soup and other cultural staple foods, also tend to contain substantial salt levels.

##### Incorporation of salt, seasonings and sauces by food operators

Food vendors and caterers acknowledged that their practice of incorporating salt, seasonings and sauces into food and even beverages played a role in influencing consumers’ Na intake. Flavour enhancers such as monosodium glutamate, stock cubes and soya sauces were commonly employed to enhance flavour profiles. The practice extended to replacing salt with these seasonings and sauces or employing them in tandem. Some food vendors also disclosed that salt was commonly added to beverages, such as mango float and *Teh Tarik*, the Malaysian version of milk tea, to enhance texture and flavour. This practice was both vendor-initiated and customer-requested, as highlighted by a caterer based in the western region of Malaysia.

##### The practice of reading nutritional labels on packaged food among food operators

When surveying food products in the market, majority of the vendors and caterers admitted that they did not have the habit of inspecting the nutritional content label. Instead, they favoured purchasing familiar products. In cases where they did seek comparisons among brands, their focus lay primarily on price labels, ingredient lists, halal certification and compliance with ‘Food Safety is the Responsibility of the Industry’ (MeSTI) standards.

#### Salt reduction policy

##### Inclusive engagement

All vendors and caterers unanimously concurred on the necessity of a salt reduction policy, emphasising the inclusion of all relevant parties, including food manufacturers. Participants stressed that manufacturers of commercial food products should actively contribute to policy development, as their products significantly contribute to the high-salt intake among consumers. A food vendor highlighted that fast food and junk food manufacturers use more salt than food vendors. Therefore, the engagement of such food manufacturers is essential in shaping the out-of-home sector’s policy.

##### Policy implementation approaches

There were different opinions on the means of policy implementation. Most food vendors and caterers opined that initiating the policy voluntarily for a preliminary period before progressing to mandatory enforcement was a feasible approach. Nonetheless, some participants believed the policy should be wholly voluntary or exclusively mandatory. They cautioned that a mandatory approach might initially face resistance from some stakeholders. However, gradual adaptation would likely transpire over time. Further, participants noted that voluntary implementation might maintain the current status quo of salt intake.

### Barriers encountered by food operators regarding salt reduction in Malaysia’s out-of-home sectors

#### Lack of a standard guideline

Most caterers and vendors highlighted the lack of guidelines for using less salt in food preparation as a significant hurdle. The absence of clear guidance makes it difficult for them to determine appropriate salt quantities and limits. Caterers and vendors also acknowledged limited knowledge regarding suitable salt substitution or reduction ingredients. In addition, they do not have the technical skills necessary to alter recipe formulations without compromising taste.

#### Challenges from consumer practices

##### Awareness of the importance of salt reduction

Most caterers pointed out that consumers were not aware and knowledgeable enough on the health importance of reducing salt in food; hence, consumers would find it hard to understand when the out-of-home sectors reduce the salt in their food. Further, they often received request from parents to add soya sauce into their kids’ meals although the food is already salty enough.

##### Consumer acceptance

Food operators are primarily concerned about consumer acceptance, given the nature of their business. Most customers complained whenever the food was bland or less salty than what they were accustomed to. Therefore, food operators must serve food according to customer preferences to avoid complaints and loss of customers.

##### Lack of consumers requests for reduced salt

In response to queries about customer requests for reduced salt, most vendors and caterers agreed that reducing salt content presents a challenge. This scenario is because most customers have never explicitly asked for their food to be less salty.

#### Food quality

##### Effect on taste, flavour and mouthfeel of reduced-salt foods and beverages

Most vendors and caterers agreed that salt reduction is not possible as that will negatively affect the taste, quality and creaminess of the foods and beverages.

##### Effect on the taste of traditional or heritage recipes

Some vendors expressed reservations about decreasing salt in traditional food or heritage recipes due to concerns that such adjustments might alter the taste and compromise such food’s customary and authentic flavours.

#### Price of regular salt

##### Comparison of regular salt prices with natural flavour enhancers and salt substitutes

Vendors and caterers highlighted that regular salt costs are significantly lower than salt substitutes such as KCl and natural flavour enhancers such as dry and fresh herbs and spices. This cost disparity encourages the prevalent use of regular salt in their culinary practices. Some vendors and caterers acknowledged that specific recipes maintain good flavours when herbs and spices are used to reduce salt. However, employing larger quantities of natural flavour enhancers incurs higher costs, as herbs and spices are more expensive than regular salt. On the other hand, since KCl is not widely available in the market, some vendors opined that it might command a higher price than regular salt. Thus, investing more in an expensive salt substitute that produces similar outcomes to regular salt might not be justified.

#### Regulation of street food vendors

##### Lack of regulatory authority

A prevailing sentiment among vendors is the need for a dedicated regulatory body, legal framework or guidelines monitoring their salt usage. They emphasised that their interactions with local authorities mainly revolve around food safety and licensing requirements, which they diligently adhere to while operating their stalls. However, no penalties or legal repercussions if their menu offerings exceed prescribed nutritional requirements.

##### The tedious license registration process

When queried about their willingness to participate in future training courses organised by MOH for preparing reduced-salt food, vendors from East Malaysia conveyed their reluctance. They cited the restrictive nature of the course, limited to registered vendors only, as the reason behind their decision. These vendors noted that many stalls are unregistered due to the complex and time-consuming procedure of obtaining a business license.

### Enablers identified by food operators regarding salt reduction in Malaysia’s out-of-home sectors

#### Enhancing knowledge among food operators

##### Establishing a standard guideline for salt reduction in food

Caterers and vendors emphasised that the availability of a clear and comprehensive guideline for reducing salt in food would greatly assist them in minimising salt usage. They proposed implementing of educational programmes designed to teach them effective methods for reducing salt in food and providing these guidelines in a precise and accessible format.

##### Implementation of mandatory courses or training

Caterers operating in the North and East Coast regions suggested that educational and awareness programmes related to salt reduction could be integrated into the existing mandatory Food Handling Course, which is required for all food operators seeking business licenses. Currently, this course only focuses on the preparation of food that is safe and hygienic.

##### Leveraging guidance from research agencies

Vendors highlighted the need for comprehensive knowledge and guidance from research and development agencies such as the Malaysian Agricultural Research and Development Institute. They emphasised the importance of such guidance to enable them to prepare reduced-salt food offerings without compromising the taste and sensory appeal.

#### Raising awareness among consumers and food operators

##### Promoting health and managing healthcare expenses as the ultimate goals

The campaign holds significant appeal for the out-of-home sectors, driven by their concern for the nation’s health. Participants also recognised the potential for reduced medical costs as an outcome of the campaign. Thus, maintaining good health and reducing medical expenses could serve as strong incentives for active participation in the policy.

##### Educating consumers on the importance and methods of salt reduction in food

Creating consumer awareness serves as a crucial pillar to bolster the endeavours of vendors and caterers in reducing salt in their offerings. These operators believe that informed customers familiar with the salt reduction campaign would understand the significance and purpose behind their efforts. Vendors suggested that educating consumers to request for lower salt content would encourage their participation in the salt reduction campaign, as they are inclined to accommodate customer preferences. Further, displaying nutritional and health information from MOH on food trucks and stalls could effectively inform customers.

##### Targeting younger consumers

Vendors and caterers stated that awareness of salt intake and its presence in food should commence at the school level. Teachers are pivotal in cultivating a culture of reduced-salt consumption in schools. Initiating this awareness from a young age could shape healthier eating habits.

#### Strategies for gaining consumer acceptance

##### Progressive salt reduction and separate salt presentation

To address consumer acceptance and mitigate the risk of losing customers, caterers and vendors suggested a gradual reduction in salt content without the customers’ knowledge. In addition, they recommended presenting salt separately on tables, allowing customers to season dishes according to their preference.

##### Introduction of reduced-salt menus

Vendors and caterers advocate for the introduction of reduced-salt menus with regular offerings. This approach provides customers with more options and can attract new patrons. Although they might lose a few customers, it would be a partial loss. Moreover, organising low-salt food festivals gains traction as a captivating method to introduce consumers to reduced-salt food by vendors. Some caterers also suggested a ‘No Salt Day’ as part of the campaign, encouraging consumers and the out-of-home sectors to prepare and consume salt-free or low salt food.

##### Promotion and recognition of health-conscious food premises

A potential government initiative involves endorsing and promoting stalls, hotels or restaurants that align with the salt reduction campaign. Recognition could be granted through certifications, labelling as a ‘Healthy Choice Stall/Caterer,’ and implementing a star-rating system. According to vendors and caterers, customers tend to be drawn to establishments with star ratings similar to those employed by hotels. Therefore, the adoption of a star-rating approach by health-conscious stalls and caterers is likely to appeal to consumers. By amplifying promotion and recognition, vendors and caterers would be encouraged to believe that their reduced-salt offerings will remain appealing to customers.

#### Food quality

##### Salt reduction through the freezing method

Vendors highlighted the challenge of reducing salt content, particularly in traditional food, due to consumer expectations of maintaining familiar flavours. However, a potential solution is to freeze the traditional food. Vendors suggest that consumers may already perceived that frozen versions might exhibit a slightly altered taste compared with the non-frozen ready-to-eat variants. This difference in taste could result in fewer complaints regarding the flavour of the frozen version.

##### Salt reduction in spices-infused dishes

A strategic approach to reducing salt intake could begin with food rich in spices and herbs for flavour. Since these ingredients contribute substantially to the overall taste, minimal additional salt is necessary. This approach aligns with reducing salt while still delivering rich and appealing flavours.

#### Regulation measures for manufactured food products

##### Enhancement of food packaging labels

Vendors and caterers suggested enhancing the packaging of high-salt food products to facilitate the identification of such items when procuring ingredients. One possible enhancement involves incorporating warning graphic labels depicting potential health risks and consequences of excessive salt consumption.

##### Control over regular salt and high-salt products distribution

Caterers suggested implementing precise regulations to govern the market for salt and high-salt products such as sauces. Such measures could contribute to curbing the availability and consumption of high-salt options.

##### Price adjustment and taxation of regular salt

Caterers in the West region suggested increasing the price of regular salt, while those in East Malaysia advocated higher government-imposed taxes on salt. These financial adjustments could discourage vendors and caterers from excessive salt usage.

##### Price adjustment of natural flavour enhancers and salt substitute

Vendors and caterers suggested reducing the cost of natural flavour enhancers and salt substitutes. This price reduction could incentivise food industry professionals, including manufacturers, to incorporate these alternatives more extensively, ultimately reducing their reliance on conventional salt.

#### Regulation of street food vendors

##### Collaborative partnership between Ministry of Health and local authorities

In the context of street food vendors, a proactive collaboration between MOH and local authorities could procure positive outcomes. This collaboration would involve monitoring and regulating the salt content and its usage according to predefined nutritional standards. Vendors stated that local authorities are currently the primary entities responsible for regularly inspecting their stalls, although primarily for licensing purposes. Expanding this partnership to encompass vigilant monitoring of vendors’ salt usage aligns with the vendors’ suggestions and is considered a practical approach.

## Discussion

Salt reduction initiatives targeting the global out-of-home sector remain limited^(21)^. Currently, these initiatives across the globe encompass a range of strategies, including interventions in settings, food reformulation with food industry, consumer education interventions, front-of-pack labelling and taxation on high-salt foods^(26)^. It is important to note that among these initiatives, only twenty-one out of seventy-four surveyed countries have focused on the formal out-of-home sector, which includes fast-food chains and restaurants, employing an approach involving intervention in these settings. Notably, these initiatives have yet to target the informal sector, specifically street food vendors. Nevertheless, several studies^(27)^, a case study-based framework^(28)^ and an informative toolkit^(29)^ have collectively provided insights into the existing challenges associated with salt reduction in this sector and enablers or suggestions to overcome them. In this qualitative study, the researchers identified specific barriers and enablers related to reducing salt content in foods within this sector, as perceived by Malaysian consumers and food operators. Although most of the research findings were similar to other similar studies reported in a comprehensive review from 2021^(27)^, this study also revealed novel barriers, such as the intricate and resource-intensive process of registering street food establishments, and the promising potential for salt reduction in dishes rich in spices and the application of the freezing method.

In the current study, consumers highlighted limited awareness and knowledge among food operators in reducing salt during cooking as a significant barrier to salt reduction. This observation supports the findings from studies conducted in the USA^(30)^ and Korea^(31)^. Food operators in this study also expressed a similar sentiment, suggesting that this lack of awareness might stem from the absence of a standardised guideline for reference. Consequently, food operators might cautiously approach salt reduction due to concerns about its potential impact on food quality. This shared concern between operators and consumers underscores the sensitivity of salt reduction and its potential to affect both food quality and consumer satisfaction unfavorably. This reluctance to adjust salt levels in food has been identified in other countries. Chefs in the USA and the UK^(32)^ and retail food sectors in India^(33)^ have similarly acknowledged their reluctance to modify salt content. This sentiment stems from the fear that altering salt levels could compromise the overall quality of the food, ultimately leading to a decrease in consumer acceptance. Such findings highlight the intricate balance that must be struck when pursuing salt reduction initiatives without compromising the culinary experience and consumer satisfaction.

Addressing this challenge could involve equipping food operators with adequate knowledge and skills for effective salt reduction through several measures. These measures encompass customised guidelines, specialised courses and training and guidance from pertinent research agencies. Similar to findings in other studies^(27)^, teaching food operators emerges as a commonly recommended enabler. In Malaysia, MOH has developed guidelines^(34–37)^ targeting the out-of-home sector to encourage healthier food preparation. However, these guidelines primarily focus on calories, fat and sugar, leaving a gap in practical guidance for reducing salt in cooking. Therefore, a comprehensive approach entails integrating practical directives to curtail salt usage into these guidelines in tandem with addressing other nutrients. Courses and training opportunities^(38–40)^ for food operators in Malaysia are already in existence. Based on a case study-based framework for healthier out-of-home food^(28)^, established programmes on food safety present a potential entry point for encouraging healthier food practices. Notably, the Food Handler Course, which mandates food safety training for all registered food operators in Malaysia, could serve as a pivotal platform. Expanding upon this foundation, the optional Healthy Catering Training^(38)^, currently emphasising low-salt food preparation, can be extended to incorporate essential nutritional components. Moreover, research agencies hold the potential to explore innovative technologies, such as freezing methods, that preserve the palatability of reduced-salt food offerings.

Insights from food operators in the current study presented several strategies to secure consumers’ acceptance of salt reduction measures. Among these suggestions are gradual salt reduction, separate salt preparation, introducing reduced-salt menus and recognising establishments that prioritise healthy food options. Valuable lessons from the successful implementation of salt reduction initiatives in the UK emphasise that gradual reductions in salt content within packaged foods often go unnoticed by taste receptors. This approach has not resulted in reported sales losses, product substitutions or increased table-side salt usage^(41–43)^. Adopting this strategy to prepare out-of-home foods could involve gradually incorporating alternative ingredients such as herbs and spices and salt substitutes^(27)^.

Further, food operators must be convinced that gradual salt reduction would not compromise consumer acceptance^(29)^. Interestingly, Malaysian food operators suggested a distinct perspective of placing salt on tables for consumers to add themselves. This suggestion contrasts with enablers from countries such as Argentina and Uruguay, where salt shakers were removed from restaurant tables, requiring patrons to request salt consciously^(44)^. Another potential strategy to gain consumer acceptance lies in introducing reduced-salt menus. According to Michael et al.^(27)^, the lack of menu and food variety can impede salt reduction in the out-of-home sector in the USA^(30)^, UK^(45)^ and Korea^(46)^, especially when many offerings are high in salt content. Therefore, creating menus designed to offer reduced-salt options could be a strategic approach to catering to consumer preferences while simultaneously promoting healthier choices.

Food operators and consumers opined that recognising healthy food establishments could significantly enhance consumer acceptance of salt reduction efforts. This recognition could manifest through certifications, labels denoting ‘healthy choice stall/caterer’ or the implementation of a star-rating system. A pertinent example of a salt reduction initiative involving labelling is the adoption of endorsement logos for Na content displayed on packaged foods. Three countries have adopted the Health Star Rating as a rating system. Meanwhile, nineteen countries, including the Netherlands and Malaysia, have integrated the Healthier Choice Logo to highlight low Na content across different brands^(26)^. Given the recommendation for a star-rating system by food operators, the Health Star Rating system from Australia and New Zealand could serve as a viable model adaptable to Malaysia’s out-of-home food sector. Concurrently, MOH has established various endorsement logos and labels voluntarily granted to food operators adhering to specified nutritional standards. For example, the BeSS certification^(37)^ is a recognition conferred upon establishments that meet criteria encompassing food safety and nutrition labelling (i.e. healthy eating poster displays, calorie tagging and the provision of plain water). Therefore, the BeSS certification could integrate salt content requirements as an initial step towards helping consumers identify health-conscious food premises as this recognition is already mandatory within government sectors, but remains voluntary in other settings. In addition, the MyChoice logo functions as an endorsement specifically for healthier menus served at respective food outlets^(47)^. This approach continues the Healthier Choice Logo program, which initially endorsed nutritious packaged food across different brands. By leveraging these existing frameworks, Malaysia’s out-of-home food industry can promote healthier choices and facilitate consumer decision-making while advancing salt reduction goals.

Lack of awareness among consumers emerged as another barrier among food operators in reducing salt content. These operators noted that consumers’ limited awareness regarding the health benefits associated with reduced-salt consumption contributed to the prevailing trend of patrons not requesting lower salt levels in their meals. On the other hand, consumers stated that the degree of saltiness in their dishes rested solely in the hands of the cooks and chefs. Despite voicing requests for reduced-salt content, they often found their meals excessively salty. This disparity in perspectives between the two groups may be linked to their distinct backgrounds, potentially contributing to the seemingly inconsistent barriers identified.

Nevertheless, this disparity highlights the significance of developing strategies to increase awareness among consumers and food operators. Consumers emphasised that the dissemination of salt-related messages remained limited. While numerous educational resources featuring context-specific salt-related information have been formulated and assessed for Malaysia, a midterm evaluation^(17)^ revealed a low public outreach and engagement. This shortfall might be attributed to inadequate federal-level mechanisms for direct communication with the public, coupled with limited opportunities and incentives at the state level to disseminate such information proactively. In addition, compared with the broader landscape of global salt reduction initiatives^(26)^, fewer countries have implemented consumer education measures in 2019 than in 2014. This decline may be due to consumer education efforts’ resource-intensive and time-consuming nature^(48,49)^. Nevertheless, based on the current findings, it is evident that consumer education remains an essential component alongside other approaches for effective salt reduction^(50–52)^. It is imperative to develop comprehensive strategies that enhance the reach and impact of these crucial messages to ensure broader outreach of salt-related messages to the public and food operators.

Challenges faced by consumers and food operators encompassed the cost and limited availability of low-salt packaged food products, the price of regular salt, natural flavour enhancers and salt substitutes. These challenges underscore the fundamental issue that food operators’ prevalent and affordable ingredients often lean towards high-salt options, while alternatives or low-salt ingredients are expensive. Notably, although commendable, Malaysia’s successful reformulation of fifty-three high-salt processed food products by 2018 had a limited impact on the population’s’ overall salt intake. This circumstance was because these reformulated foods were NS^(17)^. In contrast, the UK’s effective salt reduction strategy encompassed reformulating commonly consumed items, such as bread, dairy, meat and convenience food, which generated successful outcomes. A pivotal barrier in the Malaysian context was the delayed implementation of Na labelling regulations, which hindered meaningful engagement between food manufacturers and reformulation efforts. However, as of 2022, mandatory Na labelling has implemented after a grace period, allowing food manufacturers to update their product labels. After reformulation, it is imperative to ensure that the prices of reformulated products are accessible to a wide range of consumers, accompanied by strategic nutrition labelling. This initiative can be realised by incorporating warning graphic labels and traffic light labelling, enabling the out-of-home sector to distinguish between low- and high-salt food products. Affordable pricing is essential to incentivise the adoption of low-salt products^(40)^. The application of warning labels on front-of-pack labelling has proven effective in countries such as Chile, while eleven countries, including the UK, have implemented traffic light labelling. Accordingly, the efficacy of these enforcement measures in influencing population-wide salt intake should be vigilantly monitored, offering insights for ongoing improvement.

Consumer groups have advocated implementing mandatory salt limits for salt reduction in food. This strategic approach encourages the production of processed food items that boast reduced-salt content. Notably, fifty-seven countries have already established salt limits or targets across food products such as bread, processed meats and cheeses. However, most countries have adopted a voluntary framework for implementation^(26)^, including the UK. Hence, the researchers propose an amendment to the current Food Act 1983 to encompass salt targets across various products. This simultaneous adjustment must be balanced with an unwavering commitment to food safety, reassuring manufacturers that reformulating their products will not compromise this vital aspect^(53)^.

Consumers have expressed interest in the utilisation of salt substitutes by food manufacturers. However, it is crucial to acknowledge that certain salt substitutes, such as KCl, have demonstrated a propensity to impart a bitter taste. This aspect poses a challenge in their complete substitution for regular NaCl, commonly known as table salt^(54)^. Alternatively, natural flavour enhancers such as herbs and spices in reduced-salt food can evoke hedonic satisfaction similar to unreduced salt offerings^(55)^. Therefore, advocating for integrating these natural flavour enhancers represents a promising approach for manufacturers and out-of-home food operators. Dishes rich in spices, like curries and offerings from *Mamak* restaurants, could be selected as trial samples for salt reduction initiatives. Notably, food operators have observed that these dishes inherently require less salt. Utilising them as test cases could offer valuable insights into effective salt reduction strategies within the out-of-home food sector.

Addressing affordability in salt reduction efforts encompasses reducing the cost of low-salt food products and natural flavour enhancers to incentivise their usage within the out-of-home sector^(40)^ In addition, it includes potential interventions such as raising the price of regular salt and imposing a tax on salt. No existing salt reduction initiatives have systematically considered the price aspect for regular salt, low-salt food products or natural flavour enhancers. However, countries like Saint Vincent and Grenadines have implemented a 15 % value-added tax on salt^(26)^. Meanwhile, other countries like Fiji, Hungary, Mexico, Tonga and Thailand have introduced taxes on high-salt products. Thus, this study proposes the implementation of taxes on regular and high-salt products within the Malaysian context. However, such strategies necessitate comprehensive evaluation and analysis.

Based on the Salt Reduction Toolkit^(29)^, the WHO acknowledged the complexity of reducing salt intake within the informal out-of-home sector, particularly street food vendors, as many entities operate without registration or regulation. This challenge aligns with the perspectives shared by the vendors in the current study and a study involving local vendors in India^(33)^. In Malaysia, street vendors are only accountable to local authorities for stall registration. As part of this process, vendors must complete a Food Handling Course to ensure safe food practices, a prerequisite for registration. Further, local authorities occasionally monitor registered stalls to ensure adherence to food safety standards. However, a notable obstacle arises from the perception among vendors that the registration procedure is tedious. This perception has subsequently resulted in many vendors remaining unregistered, thereby escaping periodic supervision by the authorities.

The crux of addressing the salt reduction challenge within the informal out-of-home sector lies in the empowerment of local authorities. As highlighted in the WHO Salt Reduction Toolkit^(29)^, local authorities play a pivotal role in implementing effective strategies for salt reduction in food. This approach involves integrating nutritional considerations into the vendor registration process and establishing mechanisms for ongoing monitoring alongside food safety protocols. Despite the existing focus on food safety policies and programs in Southeast Asia, including Malaysia, it is paramount to extend this scope to include salt reduction initiatives^(28)^. For instance, vendors in Kolkata are monitored for food safety using a screening tool. As a progressive step towards promoting healthier out-of-home food options, these screening tools could be extended to encompass nutritional factors, such as weekly salt consumption. This expansion would identify vendors requiring additional training in food safety and essential nutrition. However, local authorities must establish simplified procedures for registering and licensing all vendors. This crucial step will facilitate salt content monitoring but also the vendors’ participation in future initiatives. Further, including nutrition components within the mandatory Food Handling Course, which presently centers on food safety, represents a logical progression to equip vendors with comprehensive knowledge of healthier food practices.

**Fig. 1:**
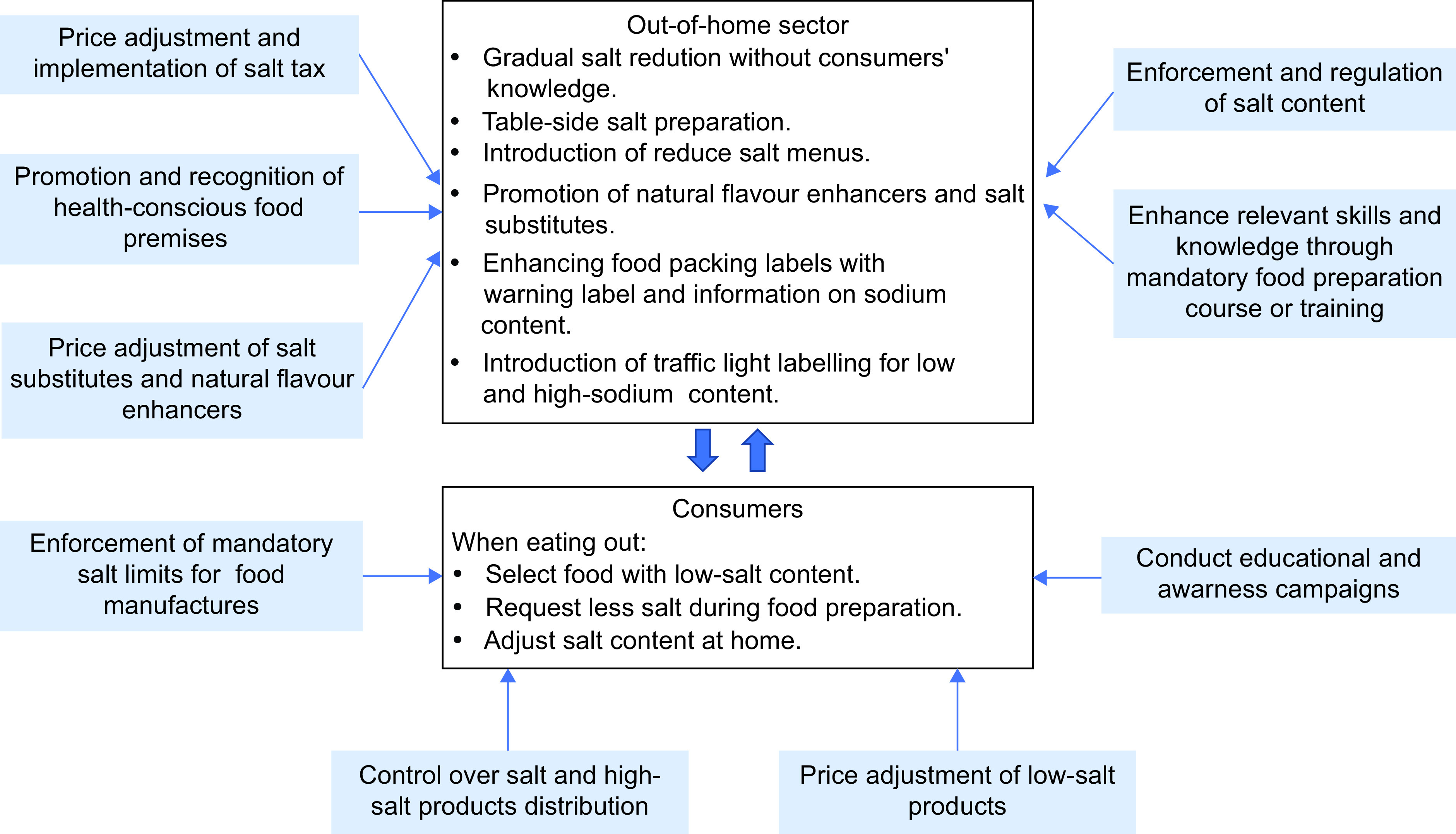
Recommended strategies to assist consumers and the out-of-home sectors towards the salt reduction policy

The active participation of food providers in the out-of-home sectors and consumers has been fundamental in enabling this study to gain diverse insights. Based on these insights, a comprehensive framework (see Fig. 1) has been devised, outlining practical strategies for salt reduction in the out-of-home food sector. This framework highlights the collaborative efforts required from consumers, the out-of-home food sector and other relevant stakeholders to foster a conducive environment for consumers to manage their salt intake when eating out. However, it is essential to acknowledge that the researchers’ presence could have influenced the input provided by participants during the sessions. Moreover, the subsequent data analysis and interpretations are inherently subject to the researchers’ skills and potential personal biases^(56)^. As such, transparency in methodology and an open recognition of potential limitations remain integral to ensuring the validity and reliability of the findings and proposed framework.

### Conclusion

In the present study, all the stakeholders agreed with the excessive salt consumption prevalent in Malaysia’s out-of-home sectors. Distinct barriers and enablers were identified within the consumer and food operator groups. Among the barriers impeding salt reduction in the out-of-home sector include concern over the undesirable effects on food quality and unsupportive practices among food operators, such as limited knowledge of salt reduction techniques and the absence of standardised guidelines. These barriers could be reduced through targeted educational initiatives for food operators. Proposed measures include the development of standardised guidelines, mandatory training courses, guidance from research agencies and strategic approaches aimed at consumer acceptance. Such strategies include gradual salt reduction, offering salt on tables for individual adjustment, introducing reduced-salt menus and promoting and acknowledging health-conscious food premises. Additional challenges include the scarcity of affordable low-salt food products and the competitive pricing of regular salt compared with natural flavour enhancers and salt substitutes. Therefore, it is essential to ensure the focus on the accessibility and affordability of healthier ingredients compared with regular salt and high-salt food products. The absence of strict enforcement mechanisms for the informal food sector poses another hurdle to successful salt reduction. Further, effective communication of salt-related messages to consumers and food operators requires enhancement, necessitating multifaceted interventions. A multifaceted intervention strategy involving out-of-home sectors and relevant stakeholders is recommended to develop a functional framework. Accordingly, the efficacy of such a framework can be gauged through outcome measurement, facilitating continual improvement.

## Supporting information

Zainal Arifen et al. supplementary material 1 Zainal Arifen et al. supplementary material

Zainal Arifen et al. supplementary material 2 Zainal Arifen et al. supplementary material

## Supplementary material

For supplementary material accompanying this paper visit https://doi.org/10.1017/S136898002300277X
