# Supplementary material for: Perceptions, barriers and enablers on salt reduction in the out-of-home sectors in Malaysia (MySaltOH) from the perspective of street food vendors, caterers and consumers
Source: Public Health Nutr. 2023 Dec 15;27(1):e12. doi: 10.1017/S136898002300277X (PMC10828668; doi:10.1017/S136898002300277X)
Supplement: Zainal Arifen et al. supplementary material 1 — Zainal Arifen et al. supplementary material [file S136898002300277Xsup001.docx]

# **APPENDIX B**

Questionnaire for consumers and street food vendors/ caterers

| No. | Items | Questions | |
| --- | --- | --- | --- |
|  |  | Consumers | Street food vendors/ Caterers |
| 1 | Societal and cultural norm and values | 1. What do you think about salt consumption in Malaysia? 2. In your opinion, generally, how much salt is used in your cooking? 3. In your understanding, what are the major food sources that are high in salt? 4. Do you use any flavor enhancers (chicken cubes, oyster sauce, *taucu*) in your cooking? 5. Do you know other alternatives or ways to increase the flavour of food without adding more salt and other flavour enhancers? 6. Do you think there is any relationship between excessive salt consumption and health? 7. In your opinion, how important do you think reducing salt intake in your diet is? 8. *What do you think about reduced salt products/ foods/ dishes? 9. *Why do you eat out? | - 1. What is your opinion on salt intake in Malaysia?   2. Is it a problem? (Why the intake is high?)   3. Do you think that high salt intake will affect the health of the population? |
| 2 | Practices and action | - 1. *Have you made an attempt to reduce your salt intake, i.e., the amount of salt you add in cooking? Or salty foods or snacks that you purchased? Or during eating out? If yes, please state the action. If no, why?   2. *When buying packaged food items, how often do you look for the nutrition information? Do you specifically look for the salt content? Do you think it would be helpful to have information about the salt content on food packages?   3. Do you currently use salt substitutes in your cooking/ dishes? Would you be willing to do so?   4. Do you use Himalayan salt, rock salt or similar salts in your cooking/ dishes? If so, why?   5. *What type of programs/ policies should be made by the government for salt reduction among the population? Media role? Food industry role? Restaurants/ hotels/ street vendors role? (e.g. Labelling sodium or salt content in food products, consumer awareness, development of symbols to identify low salt products, agreements with food industry to lower the salt content of food products, and regulations) | - 1. Can you share about your product that you sell? (Probe: What is your product that is low in salt?)   2. What is your opinion on customer/population acceptance towards low salt food?   3. Have you tried to reduce salt in your food? (Probe: Do you try modifying your recipe? Can this method be implemented?)   4. What is the support that you need to reduce salt in your food?   5. Do you add food enhancers other than salt in your product? What is the salt enhancer?   6. Do you use salt substitute in production of your product? If not, do you willing to do it? (E.g.: Potassium Chloride)   7. What is your opinion regarding salt reduction policy?   8. Does this strategy need to be voluntary or mandatory? Why? |
| 3 | Motivations | 1. *What action would encourage you to reduce the amount of salt you add in cooking? Or buying or eating out? (e.g. Media’s role, doctors’ advice, traffic light labelling system) 2. What action would encourage you to cutting habit of using salt at the table? 3. *Need to reduce your salt intake (through home prepared foods, processed foods and meals outside home)? 4. *What are other help or support do you need to reduce your salt intake (through home prepared foods, processed foods and meals outside home)? | 1. What is the factor that encourage you to participate/take action to reduce salt? 2. (If answered 2.3, don’t need to answer 3.2) Do you have a long-term target to evaluate salt reduction commitment? (Probe: How do you do it?) 3. Do you expect the salt reduction strategy as part of the social responsibility to increase health among population? |
| 4 | Barriers | 1. What would make it difficult for you to use less salt in cooking? (Family members, children’s preferences) 2. What would make it difficult for you to cutting habit of using salt/ sauces at the table? 3. *What would make it difficult for you to buy less salty foods? (Non-availability of low salt items, high price, preferences) 4. *What would make it difficult for you to choose less salt when eating out? (Family members’ preferences) | 1. How do you face all the barriers to implement salt reduction strategy/ policy? 2. Do you need support from other party to take action in you organization? (Probe: Support; knowledge/ ability/ skills to take the action?) |
| 5 | Monosodium glutamate | 1. What is your opinion on monosodium glutamate? (Probe: Bad effect, etc.) 2. Do you add in MSG in your cooking? | 1. What is your opinion on monosodium glutamate? (Probe: Bad effect, purpose of using etc.) 2. Do you add in MSG in your product? |
| 6 | Additional information | 1. *Are they any other issue that you would like to highlight in relation to salt reduction that have not already been covered? 2. Is there any effect of COVID-19 pandemic on your eating habit? 3. Does COVID-19 pandemic inhibit to enjoy eating out? Do you order food delivery? How often? What type? 4. What types of food you stock up during COVID-19 pandemic? | 1. Is there any other issue/ opinion that you would like to add? |

*Questions that were prioritized for the current study.
